# Particulate matters, aldehydes, and polycyclic aromatic hydrocarbons produced from deep-frying emissions: comparisons of three cooking oils with distinct fatty acid profiles

**DOI:** 10.1038/s41538-022-00143-5

**Published:** 2022-06-03

**Authors:** Kuang-Mao Chiang, Lili Xiu, Chiung-Yu Peng, Shih-Chun Candice Lung, Yu-Cheng Chen, Wen-Harn Pan

**Affiliations:** 1 Institute of Biomedical Sciences, Academia Sinica, Taipei, 11529 Taiwan; 2 School of Food Science and Biotechnology, Zhejiang Gongshang University, Hangzhou, 310018 China; 3 Institute of Population Health Sciences, National Health Research Institutes, Miaoli, 35053 Taiwan; 4 Department of Public Health, Kaohsiung Medical University, Kaohsiung, 80708 Taiwan; 5 Research Center for Environmental Changes, Academia Sinica, Taipei, 11529 Taiwan; 6 National Institute of Environmental Health Sciences, National Health Research Institutes, Miaoli, 35053 Taiwan; 7 Department of Occupational Safety and Health, China Medical University, Taichung, 40402 Taiwan

**Keywords:** Environmental impact, Risk factors

## Abstract

It is recognized that hazardous emissions produced from frying oils may be related to oil properties, particularly the fatty acid composition. However, investigations have been limited and partial. In this work, the emissions from deep-frying foods with three oils (palm, olive, and soybean oils) with distinct fatty acid profiles were comprehensively examined in a simulated kitchen, and the interrelationship among emitted substances, oil quality parameters, and fatty acids profiles was explored. Firstly, palm oil emitted the highest number concentration of total particle matters ((3895 ± 1796) × 10^3^ #/cm^3^), mainly in the Aitken mode (20–100 nm). We observed a positive correlation between particle number concentration and levels of palmitic acid, a major saturated fatty acid (SAFA) (r_s_ = 0.73, *p* < 0.05), and total polar compounds (TPC) (r_s_ = 0.68, *p* < 0.05) in the fried oil, a degradation marker which was also positively correlated with that of black carbon (BC) (r_s_ = 0.68, *p* < 0.05). Secondly, soybean oil emitted the highest level of gaseous aldehydes (3636 ± 607 μg/m^3^), including acrolein, propinoaldehyde, crotonaldehyde, hexanal, and trans-2-heptenal; the total aldehyde concentration were positively correlated with α-linolenic acid (ALA) percentage (r_s_ = 0.78, *p* < 0.01), while hexanal and trans-2-heptenal were with linoleic acid (LA) (r_s_ = 0.73 and 0.67, *p* < 0.05). LA and ALA were two major polyunsaturated fatty acids in non-tropical plant oils. Thirdly, palm oil emitted the most particle-bound polycyclic aromatic hydrocarbons (PAHs), and a positive association was discovered between two PAHs and SAFA percentage. Olive oil seems superior to soybean and palm oils with regards to toxic emissions during deep-frying.

## Introduction

Cooking with oils, especially deep-frying foods emits a significant amount of particulate and gaseous pollutants^1–3^. Cooking emissions have been found as one of the most important sources of organic particulate matter, contributing to 10–34% of total ambient primary organic aerosol^4,5^ and have been associated with adverse health effects, such as elevated risks of lung cancer and mutagenicity even in non-smokers^6–11^. A higher incidence of respiratory diseases in cooks has also been attributable to frequent exposure to the degradation products of cooking^12^. Various gaseous- and particle-phase toxic contaminants in the cooking emissions have been documented in the literature. Polycyclic aromatic hydrocarbons (PAHs) and aldehydes derived from pyrolysis and oxidation of organic substances are two well-recognized toxic chemical species produced during cooking^13,14^. The study also found that kitchens are an important source of black carbon (BC)^15,16^ and ultrafine particles^17^. Although the range hood is commonly used to discharge these pollutants in the kitchen, the performance of the range hood usually is not altogether satisfactory^18,19^.

Previous animal study has found that smaller particles do more damage than their larger counterparts^20^. Once these ultrafine particles are inhaled into the lung, they can travel to internal organs from the exchange region (alveolar cell) via the pulmonary vasculature and may directly injure distant organs^21^. Another potential mechanism is to spread inflammatory metabolites or mediators generated from the lung to distant organs^21^, leading to the progression of inflammatory diseases^22^.

It has been reported that deep-frying generates a higher magnitude of air pollutants than other cooking methods^1,18^. Controlled comparative studies demonstrated that the levels of PM_2.5_, PAHs, and aldehydes from cooking emissions were significantly varied by oil properties^19,23^. However, oil emissions from the deep-frying process have not been comprehensively investigated across oils with different fatty acid compositions. Therefore this study intended to compare multiple harmful emissions, either particulate or gaseous-phase contaminants, from deep-frying using three popular cooking oils with distinct fatty acid compositions. In addition, the relationship was also explored between emitted pollutant concentration and oil characteristics such as peroxide value (POV), acid value (AV), and total polar compounds (TPC) to shed lights on mechanisms.

## Results and discussions

In this study, all experiments were carried out in a standard kitchen, and the height of the range hood was installed according to the common height of ordinary Chinese families. Based on previous COF-related research, the samplers were set 15 cm beneath the range hood and 40 cm above the electric fryer to collect oil emissions. This sampling distance can minimize the effect of the turbulence from the range hood.

In addition, the flow rate of range hoods in general households is about 15 m^3^/min. In order to avoid affecting the sampling results due to the turbulence from the range hood, this experiment set a reasonable flow rate as 4 m^3^/min based on previous research.

### Real-time particle concentrations

Levels of particle mass and number concentrations, and BC concentration with real-time measurements obtained from emissions of deep-frying three cooking oils are presented in Table 1. We found the total particle number concentration emitted from palm oil ((3896 ± 1797) × 10^3^#/cm^3^) was significantly higher than that from soybean oil ((469 ± 476) × 10^3^#/cm^3^) and olive oil ((400 ± 156) × 10^3^#/cm^3^). In particular, a higher magnitude of particle number concentrations in Aitken (20–100 nm) mode and in accumulation mode (100–1000 nm) was observed for palm oil.

**Table 1:** The descriptive statistics (mean ± standard deviations) on particle mass and number concentrations, and black carbon concentration obtained from emissions of deep-frying French fries with three cooking oils.

Measurement	Soybean oil (*n* = 3)	Palm oil (*n* = 3)	Olive oil (*n* = 3)
**Mass Conc. (μg/m**^**3**^**)**			
Aitken(20–100 nm)	42.2 ± 43.8^b^	378.1 ± 180.7^a^	47.74 ± 34.2^b^
Accumulation (100–1000 nm)	1802.7 ± 982.1	2700.4 ± 861.0	2282.55 ± 1681.6
Coarse particles (>1000 nm)	4391.8 ± 1813.6	3551.4 ± 520.8	3963.80 ± 2870.6
Total particle mass conc.	6236.7 ± 2725.1	6627.9 ± 1498.6	6318.00 ± 4482.9
**Number Conc. (10**^**3**^**#/cm**^**3**^**)**			
Aitken(20–100 nm)	185.1 ± 287.5^b^	3092.8 ± 1534.4^a^	55.08 ± 364.6^b^
Accumulation (100–1000 nm)	284.0 ± 213.5^b^	802.4 ± 276.1^a^	379.76 ± 198.6^ab^
Coarse particles (>1000 nm)	0.4 ± 0.3	0.6 ± 0.2	0.46 ± 0.4
Total particle number conc.	469.6 ± 476.3^b^	3895.7 ± 1796.6^a^	400.96 ± 156.5^b^
**BC (μg/m**^**3**^**)**	0.93 ± 0.80	1.99 ± 1.01	1.74 ± 1.05

non-detectable. Presented values are mean concentrations during deep-frying French fries.

*BC* black carbon, *Conc*. concentration.

^a,b^In each row, values with different superscripts indicate significant difference at *p* < 0.05.

Although the suitability of palm oil for frying has been widely propagated due to its oxidative stability^24^, the potential health hazard from the elevated level of ultrafine particles (20–100 nm) during deep-frying should not be overlooked. Consistent with the above phenomenon, the particle number concentration was positively correlated with palmitic acid (r_s_ = 0.78, *p* < 0.05), a major SAFA in palm oil (Supplementary Table 5). Previous studies also demonstrated that SAFA emitted higher particle number concentration than PUFA and MUFA-rich oils^25–27^ and palmitic acid has been shown to be the predominant component of cooking-derived particulate matter^28,29^. Compared to PUFA or MUFA-rich oils, palm oil has a large quantity of small SAFA molecules and less polymerization to form large nonvolatile molecules during deep-frying^30^. We also found a positive correlation between the particle number concentration and total polar components (TPCs) (r_s_ = 0.68, *p* < 0.05). The TPCs refer to degraded products from the triglycerides during the deep-frying process, including fatty acids, aldehydes, ketones, alcohol, and nonvolatile products^31^. The result implies more degradation occurred in palm oil than in the other two. We also noticed that the magnitude of variation of the total number concentration of the soybean oil is relatively higher than those of palm oil and olive oil. This may be due to the fact that PUFA contains more carbon-carbon double bonds so their structures are unstable compared to SFA and MUFA^32^. Based on our experience, we would recommend to include more replicates for future studies.

**Fig. 1:**
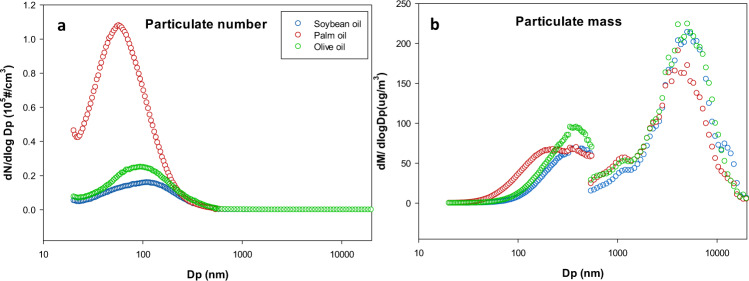
Particulate size distributions across three oils. Comparing particulate size distributions across three oils, **a** using mean* particulate number conc. generated as intensity or **b** using mean* particulate mass conc. as intensity, during the 2-h process of deep-frying French fries at 180 °C. *Mean of three repeats, each repeat provides the mean number conc. and the mean mass conc. of 120 data points (one per minute) for each oil (The blue, red, and green circles represent soybean oil, palm oil, and olive oil, respectively).

Figure 1 illustrated the particulate size distributions with respect to numbers and mass concentrations. We observed a unimodal distribution for particulate number concentration and a bimodal distribution for particulate mass concentration in all three oils. The deep-frying with palm oil, soybean oil, and olive oil produced the peak concentration of the particulate number at diameters of 57.3, 109.4, and 94.7 nm, respectively. Since similar particulate mass concentration was observed across three oils, it appears the smaller the emitted molecules the greater the particle number concentration and the larger the emitted molecules the lower the number concentration of particles. The size distribution of particles from olive oil is similar to that from other cooking oils with high oleic acid content such as the rapeseed oil^33^.

**Fig. 2:**
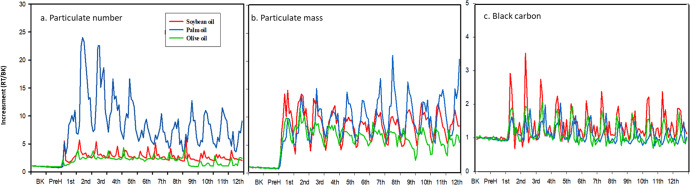
Time-series fold change of particles and black carbon concentrations emitted during deep-frying with three oils. **a** Particle number, **b** particle mass, and **c** black carbon. Y axis (RT/BK): The fold change was calculated by dividing real-time measurement levels (RT) (one per minute) by the mean background (BK) value. X axis: batch number for frying French fries. BK: 10 min-background measurements before heating oil. PreH: 10 min measurements during the preheating period before frying French fries. (The red, blue, and green lines represent soybean oil, palm oil, and olive oil, respectively).

Figure 2 shows the particle number concentrations, particle mass concentrations, and BC concentrations changed along with the 12 consecutive batches of frying. The particle number and mass concentrations as well as BC concentrations increased quickly within a few minutes and reached the peak levels for all three oils for deep-frying French fries. The mean concentration of BC generated from soybean oil, palm oil, and olive oil was 0.93 ± 0.80, 1.99 ± 1.01, and 1.74 ± 1.05 μg/m^3^, respectively (Table 1). Although the mean levels were not statistically different, a positive correlation between the average BC concentration and TPC was observed (r_s_ = 0.68, *p* < 0.05) and soybean oil peaked much higher than the other two oils (Fig. 2c). Previous studies had found that a huge amount of BC concentration may be emitted with traditional cookstoves^34,35^. Cooking with biomass solid fuels is one of the major sources of BC and the average concentration of BC ranges from 5.4 to 34.9 μg/m^3^ in household kitchens with biomass solid fuels^15,36,37^. Compared with previous studies using biomass solid fuels, BC levels (<2 μg/m^3^) in our study was much lower. It is likely due to the lower heating temperature (180 °C) and the use of an electric fryer. The positive correlations between TPC and the average particle number concentration (r_s_ = 0.68, *p* = 0.042) and between TPC and BC concentration (r_s_ = 0.68, *p* = 0.042) were observed (Supplementary Table 5). This is the first to correlate cooking emission of pollutant concentrations and oil quality indices. The correlation results may provide some clues and suggestions for further studies on reducing cooking oil emissions with respect to oil properties.

### Gaseous- and particle-phase PAHs

The gaseous- and particle-phase concentrations of 21 PAH compounds during deep-frying French fries of three oils were shown in Table 2. The total PAHs concentrations (gaseous + particle) were reported as 22.43 ± 18.62 ng/m^3^, 16.44 ± 7.09 ng/m^3^, and 7.32 ± 10.5 ng/m^3^ for soybean, palm, and olive oils, respectively. Naphthalene was the predominant gaseous-phase PAHs, accounting for 97, 62, and 87% of the gaseous-phase PAHs for soybean oil, palm oil, and olive oil, respectively. Chen et al. also found that naphthalene (67–89%) was the most abundant gaseous-phase PAHs among all exhaust samples gathered from commercial restaurants^38^. The particle-phase PAHs with high molecular weights containing 5–7 aromatic rings are toxic and carcinogenic and are considered to be hazardous substances to health^39^. Palm oil emitted significantly higher particle-phase PAHs than soybean oil and olive oil. Cyclopenta(c,d)pyrene was the predominant particle-phase PAHs, accounting for 62, 56, and 37% of the particle-phase PAH for soybean oil, palm oil, and olive oil, respectively. Benzo(a)pyrene listed as a Group 1 carcinogen by the International Agency Research on Cancer (IARC) was found in particle-bound PAHs in all three oils with concentrations of 0.039 ± 0.067, 0.11 ± 0.097, and 0.14 ± 0.023 ng/m^3^ in soybean oil, palm oil, and olive oil, respectively.

**Table 2:** The concentrations (mean ± SD) of gaseous- and particle-phase PAHs during deep-frying French fries with three cooking oils.

Compounds (ng/m^3^)	Gaseous-phase			Particle-phase			Total (Gaseous + Particle)		
	Soybean oil	Palm oil	Olive oil	Soybean oil	Palm oil	Olive oil	Soybean oil	Palm oil	Olive oi
Napthalene	21.65 ± 18.09	11.83 ± 11.07	6.31 ± 10.51	-	-	-	21.65 ± 18.09	11.83 ± 11.07	6.31 ± 10.51
Acenapthylene	-	-	-	0.06 ± 0.01	0.04 ± 0.04	0.02 ± 0.04	0.06 ± 0.01	0.06 ± 0.001	0.02 ± 0.04
Acenapthene	-	0.01 ± 0.02	-	0.01 ± 0.01	0.04 ± 0.02	0.03 ± 0.02	0.01 ± 0.01^b^	0.06 ± 0.0003^a^	0.03 ± 0.02^b^
Fluorene	-	0.22 ± 0.31	-	-	-	0.04 ± 0.01	-	0.22 ± 0.32	0.004 ± 0.01
Phenanthrene	-	1.44 ± 2.04	-	-	-	-	-	1.44 ± 2.04	-
Anthracene	0.07 ± 0.12	0.65 ± 0.53	-	-	0.0114 ± 0.0127	0.0149 ± 0.0258	0.0693 ± 0.120	0.665 ± 0.520	0.015 ± 0.026
Fluoranthene	0.07 ± 0.13	0.35 ± 0.50	-	-^b^	0.06 ± 0.03^a^	0.0052 ± 0.090^b^	0.072 ± 0.13	0.40 ± 0.46	0.0052 ± 0.01
Pyrene	0.10 ± 0.18	0.43 ± 0.30	-	0.001 ± 0.002^b^	0.06 ± 0.004^a^	0.026 ± 0.026^c^	0.11 ± 0.18	0.49 ± 0.29	0.026 ± 0.026
Benzo(a)anthracene	0.080 ± 0.012	0.099 ± 0.023	0.086 ± 0.028	0.013 ± 0.0050	0.023 ± 0.0097	0.031 ± 0.021	0.093 ± 0.011	0.123 ± 0.0093	0.116 ± 0.0074
Chrysene	0.044 ± 0.004^a^	0.064 ± 0.013^a,b^	0.035 ± 0.0012^b^	0.0018 ± 0.001^b^	0.0034 ± 0.0005^a^	0.0024 ± 0.002^a,b^	0.045 ± 0.0038^a^	0.068 ± 0.013^a,b^	0.038 ± 0.0027^b^
Cyclopenta(c,d)pyrene	-	-	-	0.26 ± 0.24^b^	0.69 ± 0.062^a^	0.30 ± 0.40^b^	0.26 ± 0.24^b^	0.72 ± 0.063^a^	0.30 ± 0.40^b^
Benzo(b)fluoranthrene	-	-	-	0.013 ± 0.011^b^	0.025 ± 0.0012^a^	0.023 ± 0.0051^b^	0.013 ± 0.011	0.025 ± 0.0013	0.023 ± 0.0051
Benzo(k)fluoranthrene	-	-	-	-^b^	0.032 ± 0.028^a,b^	0.043 ± 0.0077^a^	-^b^	0.024 ± 0.035^a,b^	0.043 ± 0.0077^a^
Benzo(e)pyrene	-	-	-	0.019 ± 0.016^b^	0.038 ± 0.0025^a^	0.030 ± 0.0047^b^	0.019 ± 0.016	0.037 ± 0.0025	0.030 ± 0.0047
Benzo(a)pyrene	-	-	-	0.039 ± 0.067	0.11 ± 0.097	0.14 ± 0.023	0.039 ± 0.067	0.082 ± 0.12	0.14 ± 0.023
Perylene	-	-	-	-	0.029 ± 0.025	0.031 ± 0.030	-	0.024 ± 0.033	0.031 ± 0.030
Dibenz(a,h)anthracene	-	-	-	-	-	-	-	-	-
Indeno(1,2,3,-cd)pyrene	-	0.20 ± 0.28	-	-	0.018 ± 0.031	0.031 ± 0.028	-	0.22 ± 0.32	0.031 ± 0.028
Benzo(g,h,i)perylene	-	-	0.072 ± 0.12	-	0.048 ± 0.084	0.0093 ± 0.016	-	0.073 ± 0.10	0.081 ± 0.12
Dibenzo(a,e)pyrene	-	-	-	-	-	-	-	-	-
Coronene	-	-	-	-	-	0.065 ± 0.11	-	-	0.065 ± 0.11
Total	22.02 ± 18.52	15.30 ± 7.10	6.51 ± 10.42	0.42 ± 0.31^b^	1.24 ± 0.16^a^	0.81 ± 0.45^a,b^	22.02 ± 18.52	15.30 ± 7.10	6.51 ± 10.42

non-detectable; Gaseous palm oil sample number = 2, sample number for all others = 3.

Mann–Whitney *U*-test was used to test the concentration difference between oils. Welch’s *t*-test was used to test the difference for gas-phase PAH.

^a,b^In each row, values with different superscripts indicate significant difference at *p* < 0.05.

The mechanisms of PAH formation has been primarily studied in combustion. Fewer studies were based on heating lipids. SAFA and palmitic acid may contribute to the formation of PAHs (Supplementary Table 7), since positive correlations were found between palmitic acid and acenaphthene (r_s_ = 0.74, *p* < 0.05) and benzo(e)pyrene (r_s_ = 0.79, *p* < 0.05) and a positive relationship was also observed between chrysene and total SAFA (r_s_ = 0.86, *p* < 0.01). A positive correlation (r_s_ = 0.72, *p* < 0.05) between benzo(k)fluoranthrene and oleic acid was found, although most PAHs were not correlated with oleic acid percentage and the total amount of PAHs emitted is very low for olive oil. These results are consistent with previous model lipids study which found methyl oleate produced more benzo(k)fluoranthrene than methyl linolenate, methyl linoleate, and methyl stearate^40^. The TPC concentrations were significantly correlated with the concentrations of acenaphthene (r_s_ = 0.83, *p* < 0.05) and benzo(e)pyrene (r_s_ = 0.79, *p* < 0.05), but there was no significant correlations between the total PAH concentration and TPC in our study, although a previous study found that TPC was significantly correlated with the concentrations of the sum of the 16 PAHs in fried oil^41^.

### Gaseous- and particle-phase aldehydes

Table 3 shows the mean concentrations of 19 gaseous- and particle-phase aldehydes during deep-frying French fries with three cooking oils. The mean concentrations of total aldehydes were significantly higher in soybean oil (3655 ± 598 μg/m^3^) than those in olive oil (2453 ± 1304 μg/m^3^) and palm oil (2197 ± 841 μg/m^3^), indicating oil rich in PUFA emitted more aldehydes than that in MUFA and SAFA. Previous studies found that oil containing more PUFA emitted more aldehydes than other oils during deep-frying^1,42^.

**Table 3:** The mean concentrations (±SD) of gaseous-and particle-phase aldehydes during deep-frying French fries with three cooking oils.

Compounds (μg/m^3^)	Gaseous-phase			Particle-phase			Total (gaseous + particle)		
	Soybean oil	Palm oil	Olive oil	Soybean oil	Palm oil	Olive oil	Soybean oil	Palm oil	Olive oil
Formaldehyde	45.9 ± 15.0	52.8 ± 19.9	56.3 ± 12.0	0.021 ± 0.017	0.0556 ± 0.0660	0.037 ± 0.029	45.9 ± 15.0	52.9 ± 19.8	56.3 ± 12.0
Acetaldehyde	118 ± 22.8	104 ± 38.8	115 ± 52.3	0.134 ± 0.176^a^	0.057 ± 0.037^a^	0.053 ± 0.065^b^	119 ± 22.6	104.2 ± 38.7	115 ± 52.3
Acetone	84.4 ± 7.23	64.6 ± 29.6	58.9 ± 24.5	0.117 ± 0.104^a^	0.021 ± 0.010^b^	0.026 ± 0.024^a,b^	84.5 ± 7.13	64.6 ± 29.6	58.9 ± 24.5
Acrolein	673 ± 110^a^	73.3 ± 61.8^b^	149 ± 123^b^	0.299 ± 0.400	0.181 ± 0.169	0.195 ± 0.158	674 ± 110^a^	73.4 ± 61.8^b^	150 ± 123^b^
Propinoaldehyde	157 ± 29.2^a^	53.1 ± 23.1^b^	94.6 ± 39.1^b^	0.101 ± 0.127	0.054 ± 0.046	0.135 ± 0.149	157 ± 29.1^a^	53.1 ± 23.1^b^	94.7 ± 39.3^b^
Crotonaldehyde	92.0 ± 15.0^a^	6.38 ± 6.46^b^	18.5 ± 21.1^b^	0.222 ± 0.221	0.155 ± 0.104	0.173 ± 0.101	92.2 ± 14.8^a^	6.53 ± 6.46^b^	18.6 ± 21.2^b^
Butryraldehyde	84.4 ± 28.5^a^	76.1 ± 30.6^a^	57.4 ± 49.7^a,b^	0.062 ± 0.058	0.137 ± 0.097	0.160 ± 0.084	84.5 ± 28.5^a^	76.2 ± 30.5^a^	57.5 ± 49.8^a,b^
Benzaldehyde	26.5 ± 45.8	-	-	2.65 ± 2.85	1.29 ± 0.742	1.20 ± 0.333	29.1 ± 44.7	1.29 ± 0.742	1.20 ± 0.333
Isovaleraldehyde	-	-	-	-	-	0.258 ± 0.262	-	-	0.258 ± 0.262
Valeraldehyde	115 ± 32	111 ± 50.7	91.1 ± 79.0	-	0.161 ± 0.279	0.155 ± 0.215	115 ± 32.0	112 ± 50.4	91.2 ± 79.1
o-Tolualdehyde	49.0 ± 26.4	11.2 ± 9.92	9.14 ± 15.8	-	-	-	49.0 ± 26.4	11.2 ± 9.92	9.14 ± 15.8
m&p-Tolualdehyde	-	-	-	-	-	-	-	-	-
Hexanal	1030 ± 207^a^	634 ± 277^b^	549 ± 477^b^	2.21 ± 2.54^a^	1.55 ± 1.81	1.50 ± 1.35	1033 ± 206^a^	636 ± 276^b^	550 ± 478^b^
2,5-Dimethylbenzaldehyde	-	8.90 ± 15.4	-	-	-	-	-	8.90 ± 15.4	-
trans-2-Heptenal	826 ± 196^a^	399 ± 162^b^	394 ± 332^b^	1.65 ± 1.68	1.70 ± 1.95	1.11 ± 0.942	828 ± 195^a^	401 ± 161^b^	395 ± 332^b^
Trans,trans-2,4-Nonadienal	61.8 ± 20.5	102 ± 89.2	114 ± 135	0.443 ± 0.369	0.355 ± 0.367	0.329 ± 0.570	62.2 ± 20.2	103 ± 89.5	115 ± 136
trans-2-Nonenal	31.7 ± 12.5^a^	17.2 ± 2.51^b^	20.2 ± 10.3^a,b^	2.63 ± 1.99	3.62 ± 3.03	2.80 ± 1.92	34.4 ± 11.2^a^	20.8 ± 1.34^b^	23.0 ± 11.2^a,b^
trans,trans-2,4-Decadienal	19.8 ± 20.1	16.4 ± 28.5	64.7 ± 63	7.17 ± 2.52	12.2 ± 8.78	9.21 ± 2.50	27.0 ± 20.9	28.6 ± 23.8	73.9 ± 60.9
Nonanal	220 ± 61.5^b^	438 ± 159^a^	637 ± 269^a^	0.837 ± 0.688^b^	5.77 ± 5.01^a^	5.61 ± 2.26^a^	222 ± 60.9^b^	444 ± 155^a^	643 ± 270^a^
**Total**	3636 ± 607^a^	2170 ± 862^b^	2430 ± 1298^a,b^	18.6 ± 12.2	27.3 ± 21.8	23.0 ± 9.94	3655 ± 598^a^	2197 ± 841^b^	2453 ± 1304^a,b^

non-detectable; Mann–Whitney *U*-test was used to test the concentration difference between oils.

^a,b^In each row, values with different superscripts indicate significant difference at *p* < 0.05.

For individual aldehydes, the top three were hexanal (1030 ± 207 μg/m^3^), trans-2-heptenal (826 ± 196 μg/m^3^), and acrolein (673 ± 110 μg/m^3^) for soybean oil; hexanal (634 ± 277 μg/m^3^), nonanal (438 ± 159 μg/m^3^), and trans-2-heptenal (399 ± 162 ug/m^3^) for palm oil; and nonanal (637 ± 269 μg/m^3^g/m^3^), hexanal (549 ± 477 μg/m^3^), and trans-2-heptenal (394 ± 332 μg/m^3^) for olive oil. Soybean oil emitted higher concentrations of aldehydes than palm oil and olive oil, in particular acrolein, propionaldehyde, crotonaldehyde, hexanal, 2-heptenal, and trans-2 nonanal in gaseous-phase and acetone in particulate-phase. Acrolein may be generated from amino acids, lipids, or carbohydrates^43^ and has been considered as a major cigarette-related lung cancer-causing agent^44,45^. While previous study results were inconsistent with respect to the potential formation of acrolein from PUFA^46–48^, the current study showed a strong relationship between acrolein and the α-linolenic acid (r_s_ = 0.72, *p* < 0.05) (Supplementary Table 6). Perilla oil which is rich in linolenic acid emitted the highest acrolein concentration during heating compared to rice bran oil, rapeseed oil, soybean oil, and sunflower oil^46^. Crotonaldehyde is also a possible human carcinogen, although there are no human carcinogenicity data^49^.

Other aldehydes also showing a strong correlation with α-linolenic acid (Supplementary Table 6) include propinoaldehyde (r_s_ = 0. 86, *p* < 0.05), crotonaldehyde (r_s_ = 0.85, *p* < 0.05), hexanal (r_s_ = 0.83, *p* < 0.05), trans-2-heptenal (r_s_ = 0.83, *p* < 0.05) and trans-2-nonenal (r_s_ = 0.78, *p* < 0.05). These aldehydes were detected higher in oils rich in linolenic acid also in previous studies^50,51^. On the other hand, hexanal (r_s_ = 0.73, *p* < 0.05) and trans-2-heptenal (r_s_ = 0.67, *p* < 0.05) showed a high correlation with linoleic acid. Nonanal was the only aldehyde which was detected in higher concentration in olive oil emission than soybean oil and palm oil and the correlation analysis indicated that the nonanal emission level was related with MUFA content in oils (r_s_ = 0.67, *p* < 0.05).

The previous study has shown that the fatty acid with more double bonds are more prone to oxidation^50^. The relative oxidation rates of methyl oleate, linoleate, and linolenate were 1: 10.3: 21.6. Hydroperoxides are the primary oxidation products first formed during the autoxidation of lipid. The hydroperoxides are quickly decomposed into various volatile compounds, including aldehydes. Our findings are consistent with the above phenomenon such that the correlation between the levels of aldehydes and PUFAs were positive, which indicated that PUFAs contribute to the generation of aldehydes during frying French fries at 180 °C.

In conclusion, fried oil fatty acids composition and oil quality are significantly correlated with particle and gas contaminants of emissions during deep-frying French fries. The main pollutants during deep-frying French fries were particulate matter and aldehydes. Soybean oil emitted the highest level of gaseous aldehydes. Palm oil emitted the most particle-bound PAHs. Olive oil seems superior to soybean and palm oils with regards to toxic emissions during deep-frying.

## Methods

### Cooking materials and procedure

**Fig. 3:**
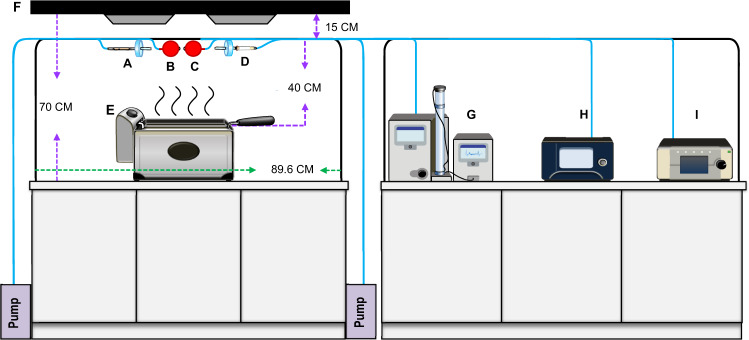
Sample collection layouts of particulate- and gas-phase cooking emissions. A: XAD-2 for gas-phase PAH samples; B: PEM for PM 2.5-PAH samples; C: PEM with DNPH-coated glass fiber filters for PM2.5-aldehyde collection; D: DNPH-coated silica cartridge for gas-phase aldehyde collection; E: Electric fryer; F: Ventilation range hood; G: SMPS; H: AE33 for real-time BC analysis; I: APS.

The experiments were conducted in a simulated kitchen (L × W × H = 3.25 m × 3.10 m × 2.75 m) at the National Health Research Institutes in Taiwan. The deep-frying process was performed in an electric fryer (5.0 L, 2000 W, L × W = 30 cm × 15.3 cm; WFT-4L, WISE Inc., Taipei, Taiwan). As shown in Fig. 3, the electric fryer was placed on the table (1.1 m above the floor) under the kitchen range hood. The hood (L × W = 89 cm × 52 cm; DR-7790ASXL, Sakura Corp., Taichung, Taiwan) was placed at 70 cm above the electric fryer (i.e., 1.8 m above the floor). We set up a backboard from the range hood to the table on the backside of the electric fryer, while front, left, and right sides of the electric fryer are opened. The default flow rate of the range hood was at 4 m^3^/min. Three popular used cooking oils, soybean oil (53.0% linoleic acid), palm oil (39.7% palmitic acid), and olive oil (72.5% oleic acid) were purchased from the local supermarket. The details on fatty acid composition, oil quality indices, and the analytical methods were provided in Supplementary Method 1 and Supplementary Table 1.

For each deep-frying process, 3.5 liters of cooking oil was used for frying consecutively 12 batches of French fries. Each batch contains 175 g of French fries (Ya Fang Inc., Taiwan). All experiments were performed following the same procedure: preheating oil for 10 min until the temperature reached 180 °C, adding French fries and frying for 8 min, and then turning off the heat for a 2-min break before the next batch. During the deep-frying process, the windows of the simulated kitchen on the side nearby the cookstove are shut to minimize the environmental draft from the outdoor. To avoid returned contaminants, we also installed the electrostatic precipitator after the range hood duct to remove the exhausted oil fume. The supply air came from the room space and outdoor.

### Sampling procedure

The concentrations of the particle number, particle mass, gaseous- and particle-phase PAHs, gaseous- and particle-phase aldehydes, and black carbon (BC) were collected from the cooking emissions of the deep-frying process. During the frying experiments, all the doors and windows were shut to avoid the inflow and environmental draft from ambient air. The sampling inlets were placed 15 cm beneath the range hood and 40 cm above the electric fryer to collect oil emissions. This sampling distance was designed to minimize the effect of the turbulence created by sucking. The kitchen air was purged by the fan for 30 min before and after the frying procedures with the door/window open. Here, we measured the particle number concentration to confirm if the concentration reach the background condition in the testing environment. For each test, the sampling process consists of 10 min-background conditions, 10 min preheating, and 120 min deep-frying (repeated 12 times) using real-time monitors and integrated samplers. The experiment was repeated three times for each cooking oil. The sampling tubes were cleaned and purged before we switched to a new testing oil.

To obtain a wide size range of particles, cooking emissions were monitored by a scanning mobility particle sizer (SMPS, TSI Inc., classifier model 3080, CPC model 3775) and an aerodynamic particle sizer spectrometer 3321(APS, TSI Inc.) for the particle number and mass concentrations. The SMPS is able to classify ultrafine particles (0.02–0.54μm) into 93 size categories and the APS can classify particles from 0.55 to 19.81 μm into 51 channels. The dominant particle size fraction and typical peak concentrations generated by different oils during deep-frying can be determined.

The particle size we measured ranged from 20.2 to 552.3 nm using SMPS and 0.523 to 19.81 μm using APS. The particle size distributions were then classified into Aitken mode (particle diameter between 20 to 100 nm), accumulation mode (particle diameter between100 to 1000 nm), and the coarse mode (>1000 nm) according to diameter^52^. The particle mass concentration was also calculated from the number concentration of the SMPS and APS using the effective density of 0.9 g/cm^3^. BC was monitored by an aethalometer (AE33, Magee Scientific, CA, USA) at the wavelength of 880 nm with a time resolution of 60 s (Supplementary Method 2).

For the PAHs, particle-phase PAHs were sampled at the flow of 10 L/min with quartz filters (37 mm, 1 μm, Whatman, UK) installed in Personal Environmental Monitor (PEM) (SKC, PA, USA) with a PM_2.5_ size and gaseous-phase was collected by a XAD-2 cartridge (SKC, Blandford, Forum, UK) at the flow of 1.0 L/min using the linear air pump (Hiblow HP150, USA). PAHs were extracted by dichloromethane and hexane mixture (2:1) and then analyzed by GC-MS/MS with multiple reaction monitoring (MRM) mode. The method detection limit (MDL) ranged from 0.63 to 2.57 ng/ml for the selected compounds (Supplementary Table 2). A known amount of PAH mixture standards was added to blank mediums through the same procedure of the analyzing samples to evaluate the recovery rate. The recovery efficiency of all compounds ranged from 73.6 to 128% (Supplementary Method 3 and Supplementary Table 2).

As for the aldehydes, particle-phase aldehydes (sampling flow rate = 10 L/min) were collected by 2, 4-DNPH-coated glass fiber filters (37 mm, 1 μm, Supelco, PA, USA) installed in PEM with a PM_2.5_ size. The gaseous-phase aldehydes were collected by a silica cartridge coated with 2, 4-DNPH (Supelco, PA, USA) after the filter sampler with a linear air pump (Hiblow HP150, USA) at a flow rate of 1.0 L/min. The flow rates of all samplers for each particle and gas sampling were adjusted using a calibration rotameter (MesaLabs Defender 520). The aldehydes in the filter and cartridge were extracted by 5 ml acetonitrile and analyzed by high-performance liquid chromatography (HPLC, PU-2089, Jasco, Japan) (Supplementary Method 4). The gradient program for HPLC aldehydes analysis is detailed in Supplementary Table 3. The MDL ranged from 0.008 to 0.058 μg/ml for the selected compounds (Supplementary Table 4). About 20 μl mixture of the aldehyde standards (10 μg/ml) was added to blank mediums (filter and silica cartridge) to evaluate the recovery efficiency. The recovery efficiency of all compounds ranged from 48.0 to 99.1% (Supplementary Table 4). When the value of PAHs and aldehydes for a given sample fell below the limit of detection (LOD) value, we assigned the corresponding sample as a value of LOD/2 for that compound.

The fatty acid composition of the fresh oils was analyzed by the Official AOAC 996.06 method^53^. Acid value (AV) and peroxide value (POV) were analyzed by official CNS 3647 N6082 and CNS 3650 N6085. Total polar compounds (TPC) were analyzed by the column chromatography method (AOAC 982.27). The detailed information on sampling, analysis methods, and QA/QC data for BC, PAHs, and aldehydes is provided in supplementary Methods.

### Statistical analysis

Non-parametric Kruskal–Wallis test was used to test whether there were significant differences among cooking emissions of three oils in concentrations of particulate matter, BC, PAHs, and aldehydes. Spearman rank correlation was used to evaluate the relationship between major fatty acid (PUFA, MUFA, or SAFA) percentage, oil quality indices (AV, POV, and TPC), and emission concentrations of the particle mass, number, and selected compounds. The time sequence curves for emission quantities of the particle mass, particle number, and BC concentrations and the correlation scatter plot were made with SigmaPlot 12.0 software (Systat Software Inc.).

## Supplementary information

Supplemental Information

## Data Availability

All data are available from the authors upon reasonable request.
