# Supplementary material for: Particulate matters, aldehydes, and polycyclic aromatic hydrocarbons produced from deep-frying emissions: comparisons of three cooking oils with distinct fatty acid profiles
Source: NPJ Sci Food. 2022 Jun 3;6:28. doi: 10.1038/s41538-022-00143-5 (PMC9166761; doi:10.1038/s41538-022-00143-5)
Supplement: Supplementary file 1 — Supplemental Information [file 41538_2022_143_MOESM1_ESM.pdf]

Supplementary information

Particulate matters, aldehydes, and polycyclic aromatic hydrocarbons produced from deep-frying emissions: comparisons of three cooking oils with distinct fatty acid profiles

1. Kuang-Mao Chiang[1,+], Lili Xiu[1,2,+], Chiung-Yu Peng[3], Shih-Chun Candice Lung[4], Yu-Cheng Chen[5,6,*], Wen-Harn Pan[1*]
2. **+ Contributed equally to this article.**
3. [1] Institute of Biomedical Sciences, Academia Sinica, Taipei 11529, Taiwan.
4. School of Food Science and Biotechnology, Zhejiang Gongshang University, Hangzhou 310018, China.
5. Department of Public Health, Kaohsiung Medical University, Kaohsiung 80708, Taiwan.
6. Research Center for Environmental Changes, Academia Sinica, Taipei 11529, Taiwan.
7. National Institute of Environmental Health Sciences, National Health Research Institutes, Miaoli 35053, Taiwan.
8. Department of Occupational Safety and Health, China Medical University, Taichung 40402, Taiwan.

List of the supplementary information:

Supplementary Method 1: Fatty acid composition and qualityof study oils.

Supplementary Method 2: The sampling procedure of particle concentration (mass/number) and black carbon in real-time.

Supplementary Method 3: Integrated PAHs sampling and analysis method.

Supplementary Method 4: Integrated aldehydes sampling and analysis method.

Supplementary Table 1:Fatty acid composition profiles of soybean oil, palm oil, and olive oil before and after deep-frying for 2 hours.

Supplementary Table 2: The retention time (RT), MRM transitions, calibration curve, LOD, MDL, and recovery rate for target PAHs.

Supplementary Table 3. The gradient program for aldehydes analysis in the HPLC.

Supplementary Table 4. The retention time (RT), reproducibility, calibration curve, LOD, LOQ, MDL, and recovery rate for target aldehydes.

Supplementary Table 5. Spearman correlation between cooking pollution and oil characteristics.

Supplementary Table 6. Spearman correlation between aldehyde and oil characteristics.

Supplementary Table 7. Spearman correlation between total PAHsa and oil characteristics.

Supplementary Method 1: The study oils fatty acid composition and quality analysis and results.

Fatty acid composition of the fresh oils was analyzed by Official AOAC 996.06 method[1]. Acid value (AV) and peroxide value (POV) were analyzed by official CNS 3647 N6082 and CNS 3650 N6085. Total polar compounds (TPC) were analyzed by column chromatography method (AOAC 982.27).

The results showed that soybean oil consists of 61.5% PUFA (linoleic acid 53.0%), olive oil is rich MUFA (73.88, oleic acid 72.5%) and palm oil contains abundant SFA (45.68%, palmitic acid 39.7%). There were slightly changes in fatty acid composition after deep-frying at 180℃ for 2 hours (Table S1). The amount of TPC, AV and POV in soybean, palm and olive oils increased after deep-frying French fries, and soybean oil had the highest increments in TPC and POV, meaning that the fatty acids in soybean oil suffered the highest level of oxidation and degradation. Nonetheless, the content of TPCs and AV did not exceed the maximum legislative limit of 25% and 2.0 mg KOH/g of Taiwan Food and Drug Administration (FDA).

Supplementary Method 2: The sampling procedure of particulate concentration (mass/number) and black carbon in real-time.

The particle number concentration monitored by scanning mobility particle sizer (SMPS) was calculated using the following equation:

$$n = \frac{c}{tQ} * \frac{\emptyset}{\eta} \qquad (1)$$

where n is number weighted concentration per channel. c is total particle counts. t is sample time. Q is sample flow rate. $\emptyset$ is sample dilution factor. η is sample efficiency factor per channel.The particle mass concentration was calculated using the following equation:

$$m = \rho V \qquad (2)$$

m is mass weighted concentration per channel. ρ is particle density. V is total volume concentration.

$$\frac{dN}{d\log Dp} = \sum_{i=1}^{n} \frac{N_i}{(\sqrt{2\pi})\log\sigma_i} \exp\left(-\frac{(\log D_P - \log \bar{D}_{P,i})^2}{2(\log\sigma_i)^2}\right) \qquad (3)$$

where N_i is the number concentration, $\bar{D}_{P,i}$ is the geometric mean diameter, and σ_i is the standard deviation of the ith log-normal mode.

All the calculations were performed by Aerosol Instrument. Manager® Software.

Supplementary Method 3: Integrated PAHs analysis method.

The PM2.5-bound PAHs sampled in quartz filters were extracted at 100 °C by

25 ml of dichloromethane and hexane mixture (2:1) in the MARS Xpress microwave digestion system for 15 min. The extract was then concentrated to about 0.8 ml by auto vacuum nitrogen blowing concentrators (Horizon Technology Inc Salem, XcelVap) and then purified with solid-phase extraction (ProElut^{TM} Bap extraction cartridge, CA, USA). The hexane eluent was concentrated to about 0.5 ml and reconstructed to 1 ml with hexane after adding 100 ul mixture of internal standards (Naphthalene-d_8, Acenaphthene-d_{10}, Phenanthrene-d_{10}, Chrysene-d_{12}, Peryleene-d_{12}).

Gas Chromatograph (Agilent Technologies 7890B) coupled with mass spectrometric detection (Agilent Technologies 7000C MS-MS triple Quard) was used for analysis. The capillary column (Agilent 122-9632 DB-EUPAH, 0.25 mm (internal diameter) × 30 m (length) × 0.25 um (film thickness)) was used for compounds separation. Helium was used as a carrier gas at a constant flow rate of 54.19 ml/min. GC-MS/MS was operated under the following conditions: oven temperature program was held for 1 minute at 80 °C, elevated to 200 °C with a speed of 25 °C/min, and then to 335 °C with a speed of 8 °C/min (hold 17.325 min). In GC-MS/MS with MRM mode, each compound was positively identified by retention time, quantifier product ion and one qualifier product ion. The optimized MRM transitions with collision energies were listed in Table S2.

Supplementary Method 4: Integrated aldehydes sampling and analysis method.

The flow rate of sampling was set at 10 L/min for particulate-phase aldehydes and 1 L/min for gaseous-phase aldehydes adsorption at the beginning and the flow rate was measured after sampling was completed. The average of these two values was multiplied by the sampling time (130 min) to determine the total volume of air sampled during each oil frying session. After sampling, the cartridges/filters were closed with endcaps/lids, sealed with paraffin and stored at -20 °C until analysis. The aldehydes analysis method is described in previous study.[3] In brief, aldehydes derivatives were extracted by acetonitrile (5 ml) for 30 min and analyzed by high-performance liquid chromatography (HPLC, PU-2089, Jasco, Japan) combined with an ultraviolet detector (UV, Varian ProStar 320, Varian, USA). The detection wavelength was 360 nm. The chromatographic separation was carried out in a reverse-phase column (Ascentis® RP-Amide Column, 5 µm, 250 × 4.6 mm, Supelco, Bellefonte, Pennsylvania, USA) with a gradient mobile phase of acetonitrile/water. The gradient program for aldehydes analysis in the HPLC is listed in Table S3. Table S4 provides the results of quality assurance and quality control of methods. The method was quantitatively characterized with acceptable reproducibiltity (relative standard deviation (RSD) < 11%), and recovery (48%-99.1%).

Supplementary Table 1. Fatty acid composition profiles of soybean oil, palm oil, and olive oil before and after deep-frying for 2 hours.

	Fresh oils (n=1)			Fried oils (mean, n=3)		
	Soybean oil	Palm oil	Olive oil	Soybean oil	Palm oil	Olive oil
**Fatty acid(g/100g)**						
Butyric acid(C 4:0)	-	-	-	-	-	-
Caproic acid(C 6:0)	-	-	-	-	-	-
Caprylic acid(C 8:0)	-	-	-	-	-	-
Capric acid(C 10:0)	-	-	-	-	-	-
Undecanoic acid(C 11:0)	-	-	-	-	-	-
Lauric acid(C 12:0)	-	0.19	-	-	0.19	-
Tridecanoic acid(C 13:0)	-	-	-	-	-	-
Myristic acid(C 14:0)	0.07	0.98	-	0.08	0.96	-
Myristoleic acid(C 14:1)	-	-	-	-	-	-
Pentadecanoic acid(C 15:0)	-	-	-	-	-	-
cis-10-Pentadecanoic acid(C 15:1)	-	-	-	-	-	-
Palmitic acid(C 16:0)	11.0	39.7	12.6	10.9	39.4	12.7
Palmitoleic acid(C 16:1)	0.08	0.18	1.10	0.36	0.18	1.12
Margaric acid(C 17:0)	0.10	0.09	0.09	0.11	0.09	0.09
cis-10-Heptadecenoic acid(C 17:1)	-	-	-	-	-	-
Stearic acid (C18:0)	4.29	4.15	2.57	4.31	4.11	2.56
Oleic acid(C18:1)	21.9	42.6	72.5	22.9	43.0	72.2

Linoleic acid (C18:2) (ω-6)	53.0	11.1	9.7	52.2	11.0	9.75
α-Linolenic acid(α -C 18:3) (ω-3)	8.47	0.29	0.60	8.35	0.42	0.71
γ-Linolenic acid(γ -C 18:3) (ω-6)	-	-	-	-	-	-
Conjugated Linoleic acid(C 18:2 con)	-	-	-	-	-	-
Arachidic acid (C 20:0)	0.35	0.38	0.43	0.35	0.38	0.43
Gadoleic acid (C 20:1)	0.16	0.15	0.27	0.18	0.17	0.28
Eicosadienoic acid (C 20:2)	-	-	-	0.05	-	-
cis-8,11,14-Eicosatrienoic acid(C 20:3)(ω-6)	-	-	-	-	-	-
cis-11,14,17-Eicosatrienoic acid(C 20:3)(ω-3)	-	-	-	-	-	-
Arachidonic acid (C 20:4)(ω-6)	-	-	-	-	-	-
Arachidonic acid(C 20:4)(ω-3)	-	-	-	-	-	-
Eicosapentaenoic acid (C 20:5)(ω-3)	-	-	-	-	-	-
Heneicosanoic acid (C 21:0)	-	-	-	-	-	-
Behenic acid (C 22:0)	0.37	0.07	0.14	0.38	0.07	0.14
Erucic acid (C 22:1)(ω-6)	-	-	-	-	-	-
Docosadienoic acid (C 22:2)(ω-6)	-	-	-	-	-	-
Docosatetraenoic acid (C 22:4)(ω-6)	-	-	-	-	-	-
Docosapentaenoic acid (C 22:5)(ω-6)	-	-	-	-	-	-
Docosapentaenoic acid (C 22:5)(ω-3)	-	-	-	-	-	-
Docosahexaenoic acid (DHA) (C 22:6)(ω-3)	-	-	-	-	-	-
Tricoasnoic acid (C 23:0)	-	-	-	-	-	-
Lignoceric acid (C 24:0)	0.11	0.08	0.06	0.13	0.08	0.06
Nervonic acid (C 24:1)	-	-	-	-	-	-

MUFA	22.2	43.0	73.9	23.2	43.3	73.6
PUFA	61.5	11.4	10.3	60.6	11.4	10.5
SFA	16.3	45.7	15.9	16.2	45.2	15.9
**Oil quality index**						
TPC (g/100g)	2.88	6.94	3.76	5.14	8.14	6.17
AV(mg KOH/g)	0.06	0.05	0.04	0.12	0.144	0.14
POV(meq/kg)	1	0.32	4.86	9.44	2.814	7.60

- :Non-detectable.The fatty acid con

Supplementary Table 2. The retention time (RT), MRM transitions, calibration curve, LOD, MDL, and recovery rate for target PAHs.

Chemical	RT	1st Collision		2nd Collision		Calibration curve		LOD	MDL	Recovery rate
		Parent (product) ion	Energy	Parent (product) ion	Energy					
	(min)	(m/z)	(eV)	(m/z)	(eV)	Slope	R^2	ng/mL	ng/mL	(%)
Napthalene	5.82	128(102)	20	128(127)	22	0.0124	0.9981	0.12	2.57	128
Acenapthylene	7.89	152(150)	40	152(151)	40	0.0088	0.9995	0.45	0.63	79.2
Acenapthene	8.07	154(152)	40	153(152)	40	0.0197	0.9981	0.38	0.84	88.0
Fluorene	8.85	166(165)	30	166(163)	34	0.0387	0.9993	0.17	0.92	85.8
Phenanthrene	11.07	178(176)	34	178(176)	34	0.0238	0.9991	0.06	0.74	98.0
Anthracene	11.13	178(176)	34	178(176)	34	0.0241	0.9998	0.16	0.77	89.4
Fluoranthene	14.25	202(200)	50	202(201)	30	0.0418	0.9993	0.13	1.04	92.6
Pyrene	15.10	202(200)	50	202(201)	50	0.1121	0.9998	0.22	0.65	92.7
Benzo(a)anthracene	18.63	228(226)	38	113(112)	15	0.1086	0.9988	0.25	0.79	97.2
Chrysene	18.89	228(226)	38	228(224)	38	0.1235	0.9990	0.18	0.87	94.9
Cyclopenta(c,d)pyrene	18.90	226(224)	50	228(224)	38	0.045	0.9981	0.13	0.68	96
Benzo(b)fluoranthrene	21.93	252(250)	42	250(248)	40	0.149	0.9989	0.17	0.92	92.7
Benzo(k)fluoranthrene	21.99	252(250)	42	250(248)	40	0.1548	0.999	0.31	1.19	93.5
Benzo(e)pyrene	22.99	252(250)	42	250(248)	40	0.2381	0.9995	0.14	0.94	93.4
Benzo(a)pyrene	23.15	252(250)	42	250(248)	40	0.1772	0.9984	0.16	1.02	91.5
Perylene	23.51	252(250)	40	250(248)	40	0.1971	0.9995	0.33	0.98	91.1

Dibenz(a,h)anthracene	26.72	278(276)	38	277(275)	38	0.1163	0.9999	0.16	0.79	81.5
Indeno(1,2,3,-cd)pyrene	26.78	276(274)	42	274(272)	42	0.0662	0.9997	0.11	1.01	82.0
Benzo(b)chrysene	27.25	278(276)	50	279(277)	45	0.0965	0.9996	0.23	0.87	73.6
Benzo(g,h,i)perylene	28.25	274(272)	42	274(272)	42	0.0158	0.9996	0.21	0.76	84.1
Dibenzo(a,e)pyrene	36.69	300(298)	45	277(275)	38	0.0325	0.9997	0.21	0.86	79.2
Coronene	37.33	300(298)	55	150(149)	20	0.0947	0.9995	0.23	0.68	94.2

RT: retention time; LOD: limit of detection; MDL: method detection limit.

Supplementary Table 3. The gradient program for aldehydes analysis in the HPLC.

Time (min)	Acetonitrile (%)	H2O (%)
0	40	60
1	40	60
25	65	35
30	65	35
58	95	5
61	95	5
71	40	60
73	40	60

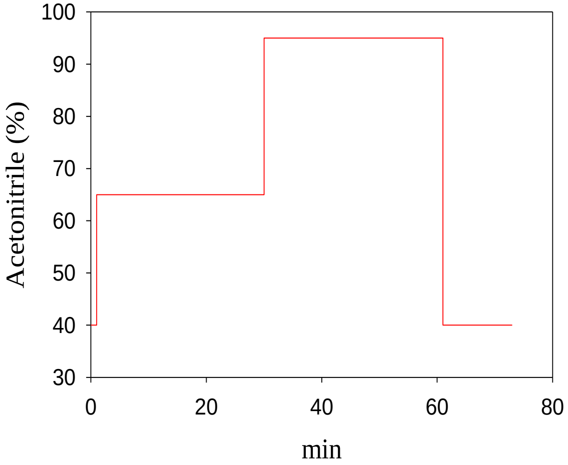

Supplementary Table 4. The retention time (RT), reproducibility, calibration curve, LOD, LOQ, MDL, and recovery rate for target aldehydes.

Chemical	RT	Reproducibility (%)	Calibration curve		LOD	LOQ	MDL	Recovery rate	
	(min)	Avg.	Slope	R^2		μg/mL		Gaseous	Particulate
Formaldehyde	13.36	0.5%	1259433	0.99962	0.003	0.008	0.008	53.67%	73.78%
Acetaldehyde	16.96	4.2%	815917	0.99963	0.003	0.012	0.011	50.82%	59.92%
Acrolein	20.26	0.7%	718729	0.99979	0.006	0.021	0.011	47.98%	46.07%
Propinoaldehyde	21.18	0.5%	924083	0.9998	0.006	0.023	0.013	47.98%	46.07%
Crotonaldehyde	22.32	0.5%	686073	0.99979	0.015	0.045	0.021	53.48%	52.46%
Butyraldehyde	26.82	0.7%	573088	0.99978	0.010	0.034	0.016	53.48%	52.46%
Benzaldehyde	29.37	0.6%	441117	0.99979	0.012	0.039	0.016	56.41%	76.31%
Isovaleraldehyde	31.59	0.9%	488898	0.99976	0.007	0.025	0.014	51.11%	67.01%
Valeraldehyde	33.77	0.5%	346234	0.9998	0.013	0.044	0.027	51.11%	67.01%
o-Tolualdehyde	34.33	0.8%	761937	0.99981	0.011	0.039	0.021	65.37%	71.71%
m&p-Tolualdehyde	37.22	1.0%	416275	0.99979	0.007	0.024	0.016	65.37%	71.71%
Hexaldeyde	40.51	1.1%	333661	0.99925	0.008	0.028	0.019	65.37%	71.71%
2,5-Dimethylbenzaldehyde	45.99	1.7%	190927	0.99914	0.010	0.036	0.027	65.37%	71.71%
trans-2-Heptenal	48.80	3.2%	291447	0.99943	0.017	0.060	0.041	96.96%	82.79%
trans,trans-2,4-Nonadienal	49.58	5.4%	124777	0.99894	0.038	0.121	0.072	99.03%	95.69%
trans-2-Nonenal	50.12	3.0%	255965	0.99941	0.012	0.046	0.030	98.03%	84.28%
trans,trans-2,4-Decadienal	52.25	1.9%	113295	0.99935	0.026	0.083	0.058	93.20%	93.78%
Nonanal	55.29	3.2%	144492	0.9993	0.025	0.088	0.042	86.68%	95.12%

RT: retention time; LOD: limit of detection; LOQ: limit of quantitation; MDL: method detection limit.

Supplementary Table 5. Spearman correlation between cooking pollution and oil characteristics.

	Particle number	Particle mass	Black carbon
PUFA	-0.12	0.17	-0.22
Linoleic acid	-0.09	0.10	-0.27
α-Linolenic acid	-0.79	0.32	-0.26
MUFA	0.13	-0.12	0.27
Oleic acid	0.13	-0.12	0.27
Palmitoleic acid	0.09	0.09	0.53
SAFA	0.73*	0.07	0.00
Palmitic acid	0.78*	0.02	0.40
Stearic acid	-0.08	-0.17	-0.44
TPC	0.68*	0.12	0.68*
AV	0.24	0.51	0.39
POV	-0.46	-0.11	-0.70

* $p<0.05$,** $p<0.01$, *** $p<0.001$

Supplementary Table 6. Spearman correlation between aldehydes [a] and oil characteristics.

	Acrolein	Propinoaldehyde	Crotonaldehyde	Hexaldehyde	trans-2-Heptenal	trans-2-Nonenal	Nonanal	19 aldehydes
**PUFA**	0.48	0.55	0.57	0.7667*	0.7000*	0.53	-0.7167*	0.55
Linoleic acid	0.47	0.51	0.54	0.7280*	0.6695*	0.49	-0.7448*	0.51
α-Linolenic acid	0.7197*	0.8619**	0.8536**	0.8285**	0.8285**	0.7782**	-0.50	0.7782**
**MUFA**	-0.52	-0.58	-0.58	-0.7833*	-0.7333*	-0.55	0.6667*	-0.58
Oleic acid	-0.52	-0.58	-0.58	-0.7833*	-0.7333*	-0.55	0.6667*	-0.58
Palmitoleic acid	-0.44	-0.14	-0.25	-0.29	-0.21	0.01	0.7576*	0.01
**SAFA**	-0.08	-0.25	-0.13	-0.05	-0.07	-0.55	-0.18	-0.15
Palmitic acid	-0.6833*	-0.7000*	-0.6833*	-0.7000*	-0.6667*	-0.55	0.62	-0.55
Stearic acid	0.47	0.36	0.43	0.61	0.54	0.01	-0.8536**	0.30
**TPC**	-0.7667*	-0.60	-0.75*	-0.57	-0.6833*	-0.55	0.60	-0.53
**AV**	-0.12	0.34	0.25	0.20	0.37	0.59	0.66	0.61
**POV**	0.60	0.56	0.56	0.37	0.44	0.33	-0.36	0.36

[a] Aldehydes which had significant different between three oils.

* $p<0.05$,** $p<0.01$, *** $p<0.001$

Supplementary Table 7. Spearman correlation between total PAHs[a] and oil characteristics.

	Acenapthene (total)	Fluoranthene (particle)	Pyrene	Chrysene	Cyclopenta (c,d)pyrene	Benzo(b) fluoranthrene	Benzo(k) fluoranthrene	Benzo(e) pyrene	21 PAHs
**PUFA**	-0.50	0.27	0.06	0.60	-0.19	-0.52	-0.65	-0.26	0.38
Linoleic acid	-0.52	0.24	0.04	0.57	-0.25	-0.57	-0.69	-0.31	0.43
α-Linolenic acid	-0.8333**	-0.23	-0.35	-0.17	-0.37	-0.62	-0.36	-0.69	0.05
**MUFA**	0.52	-0.22	0.01	-0.57	0.17	0.50	0.7610*	0.24	-0.45
Oleic acid	0.52	-0.22	0.01	-0.57	0.17	0.50	0.72*	0.24	-0.45
Palmitoleic acid	0.17	0.02	0.29	-0.53	0.10	0.27	0.67	0.22	-0.34
**SAFA**	0.24	0.44	0.28	0.8571**	0.23	0.10	-0.42	0.43	0.29
Palmitic acid	0.7381*	0.29	0.42	0.21	0.37	0.60	0.46	0.7857*	-0.21
Stearic acid	-0.38	0.09	-0.13	0.54	-0.19	-0.44	-0.7401*	-0.30	0.37
**TPC**	0. 8333*	0.39	0.55	0.26	0.37	0.60	0.49	0.7857*	-0.17
**AV**	-0.63	-0.49	-0.39	-0.51	-0.8836**	-0.8295*	-0.31	-0.81	0.24
**POV**	-0.21	-0.56	-0.54	-0.6190*	-0.02	0.07	0.20	-0.3333*	-0.50

[a] PAHs (gasous+particle) which had significant different between three oils.

* $p<0.05$,** $p<0.01$, *** $p<0.001$

REFERENCES

1. Shin, J. M.; Hwang, Y. O.; Tu, O. J.; Jo, H. B.; Kim, J. H.; Chae, Y. Z.; Rhu, K. H.; Park, S. K., Comparison of different methods to quantify fat classes in bakery products. *Food Chem* **2013,** *136* (2), 703-709.
2. Childers, J. W.; Witherspoon, C. L.; Smith, L. B.; Pleil, J. D., Real-time and integrated measurement of potential human exposure to particle-bound polycyclic aromatic hydrocarbons (PAHs) from aircraft exhaust. *Environ Health Persp* **2000,** *108* (9), 853-862.
3. Peng, C. Y.; Lang, C. H.; Lin, P. C.; Kuo, Y. C., Effects of cooking method, cooking oil, and food type on aldehyde emissions in cooking oil fumes. *J Hazard Mater* **2017,** *324*, 160-167.
